# Rational design of cholesterol oxidase for efficient bioresolution of cholestane skeleton substrates

**DOI:** 10.1038/s41598-017-16768-6

**Published:** 2017-11-27

**Authors:** Hui-Min Qin, Zhangliang Zhu, Zheng Ma, Panpan Xu, Qianqian Guo, Songtao Li, Jian-Wen Wang, Shuhong Mao, Fufeng Liu, Fuping Lu

**Affiliations:** 1 State Key Laboratory of Food Nutrition and Safety, Tianjin, P.R. China; 2 Key Laboratory of Industrial Fermentation Microbiology, Ministry of Education, Tianjin, P.R. China; 3 Tianjin Key Laboratory of Industrial Microbiology, Tianjin, P.R. China; 4 College of Biotechnology, Tianjin University of Science and Technology, Tianjin, P.R. China; 5 National Engineering Laboratory for Industrial Enzymes, Tianjin, 300457 P.R. China

## Abstract

Cholesterol oxidase catalyzes the oxidation and isomerization of the cholestane substrates leading to the addition of a hydroxyl group at the C3 position. Rational engineering of the cholesterol oxidase from *Pimelobacter simplex* (PsChO) was performed. Mutagenesis of V64 and F70 improved the catalytic activities toward cholestane substrates. Molecular dynamics simulations, together with structure-activity relationship analysis, revealed that both V64C and F70V increased the binding free energy between PsChO mutants and cholesterol. F70V and V64C mutations might cause the movement of loops L56-P77, K45-P49 and L350-E354 at active site. They enlarged the substrate-binding cavity and relieved the steric interference with substrates facilitating recognition of C17 hydrophobic substrates with long side chain substrates.

## Introduction

Cholesterol is the precursor of the five major classes of steroid hormones: glucocorticoids, mineralocorticoids, androgens, estrogens, and progestogens, which mediate a wide variety of developmental and physiological events in multicellular organisms^1–3^. It is also a frequently determined analyte in clinical analysis, diagnosis and prevention of many clinical disorders^4^. On the other hand, cholesterol could be used by mycobacteria as carbon and energy source to transform sterols into highly valuable sterol drug intermediates^5^. For instance, 4-cholesten-3-one, as cholesterol metabolite and analog, could be a potential candidate for anti-metastasis of lung adenocarcinoma^6^.

Cholesterol oxidase (ChO, EC 1.1.3.6), a bifunctional oxidase flavoprotein, catalyzes the dehydrogenation and the isomerization of the hydroxyl group at C3 position (C3-OH) of a cholestane system to yield the corresponding carbonyl product^7^. It plays a critical role in sterol catabolism by converting 3β-hydroxy steroids to 3-oxo-4-ene steroids^8^. In addition, ChO can be used for biosynthesis of steroid hormones and other pharmaceutical steroids^9,10^. This enzyme is also known as a virulence factor in pathogenic species, i.e., *Mycobacterium* sp.^11^ and *Rhodococcus equi* ^12^. ChO has emerged as a useful biotechnological tool employed for the determination of serum and food cholesterol levels^13^. Protein engineering provides a rigorous strategy and allows the creation of novel enzymes for biotechnological applications^14,15^. Rational design based on structure and function relationships has achieved significant success for improving enzymatic performance^16,17^. For instance, Toyama and co-workers introduced amino acid substitutions into *Streptomyces* cholesterol oxidase using site-directed mutagenesis and structural comparisons to investigate substrate specificity and affinity^18^. A mutant of Q145E substitution from *R. equi* enzyme showed improved thermal stability^19^. *Brevibacterium* sp. cholesterol oxidase was engineered to improve the thermal stability and activity based on the structural analysis, whereas Q153E/F128L mutant showed superior thermal stability and enzymatic activity than wild type enzyme^20^. The rational design of cholesterol oxidase (ChO) variants for its potential industrial application as an enzyme electrode for measuring total serum cholesterol is challenging.

**Figure 1:**
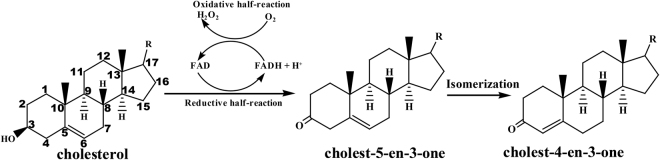
PsChO catalyzes the dehydrogenation at C3-position of cholestane skeleton substrates.

Cholesterol oxidases from various microorganisms have been subjected to protein engineering and considerable attention is devoted to cholesterol oxidase for its biotechnological applications. Recently, *Pimelobacter simplex* (also called *Arthrobacter simplex*) is widely used in the steroids transformation for its high tolerance to organic solvents^21^, and a novel putative cholesterol oxidase gene from *Pimelobacter simplex*, PsChO, was identified^22^. PsChO is a novel flavin-dependent enzyme that catalyzes the dehydrogenation at C3-position of cholestane skeleton substrates (Figs 1 and S1). However, enzymatic activity and substrate selectivity remain poorly understood, and this has hampered its application toward industrial catalyst. Therefore, successful rational design of functional PsChO would greatly advance our understanding of this family of enzymes. Moreover, insight into substrate selectivity of PsChO can be achieved by structural comparison of the wild-type (WT) and mutants using molecular simulations (MD). To obtain an enzyme with higher catalytic activity, molecular modeling combined with mutagenesis and biological assays were used to obtain a PsChO mutant with better substrate specificity and activity.

## Results and Discussion

### Structural analysis of PsChO

**Figure 2:**
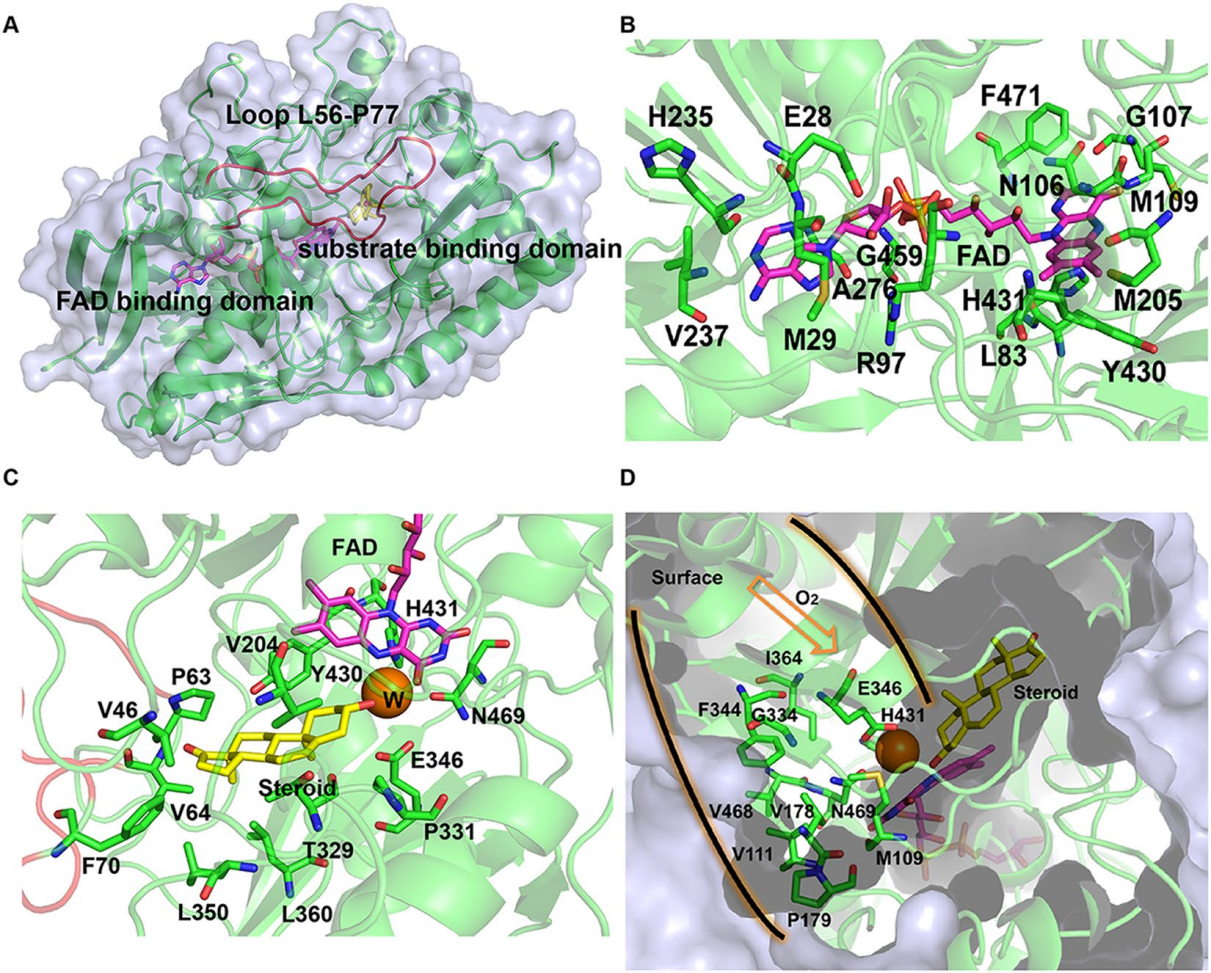
Structural analysis of the PsChO-substrate complex. (**A**) Ribbon representation of the PsChO overall structure. The loop colored red stands is the flexible lid (L55-P77). FAD and the substrate dehydroepiandrosterone are shown as magenta and yellow stick, respectively. (**B**) Interaction in the cofactor-binding site between FAD (magenta) and PsChO (green sticks). (**C**) The predicted substrate binding model. The substrate analogue is shown as yellow stick. The orange sphere stands for active water molecular. (**D**) The oxygen channel proposed to function in access of dioxygen.

We generated a structural homology model of PsChO using *Brevibacterium sterolicum* cholesterol oxidase as template (PDB ID: 1COY, sequence identity 66%, root-mean-square deviation (RMSD), 0.06 Å; 488 aligned C_α_ atoms)^23^, which possessed the characteristic nucleotide-binding fold (Rossmann fold) consisting of a β-pleated sheet sandwiched between α-helices (Fig. 2A). A loop region (L56-P77), which is located at the entrance of the active-site cavity, forms a flexible lid^23,24^. This flexible loop is presumed to open and allow the substrate to enter the binding site and then close to seal steroid from the solvent^25,26^. The substrate-binding domain shows a variety of different topologies in ChO family (Figs S2 and S3). PsChO contains a topologically equivalent FAD binding domain^24,25^, in which partially conserved residues E28, M29, L83, R97, N106, G107, M109, M205, H235, V237, A276, Y430, F471 and G459 may form hydrogen bonds or hydrophobic interactions with the cofactor FAD (Fig. 2B). The internal cavity is occupied by a lattice of nonconserved hydrophobic residues: V46, P63, V64, F70, T329, P331, L360 and Y430 (Fig. 2C). The substrate is buried within this cavity and these residues are predicted to interact with the substrate. A narrow-gated oxygen channel, which is composed of M109, V111, V178, P179, G334, F344 and V468, plays an important role in oxygen entry to the active site from the surface during flavin reoxidation (Fig. 2D).

### Characteristics of the active site

**Figure 3:**
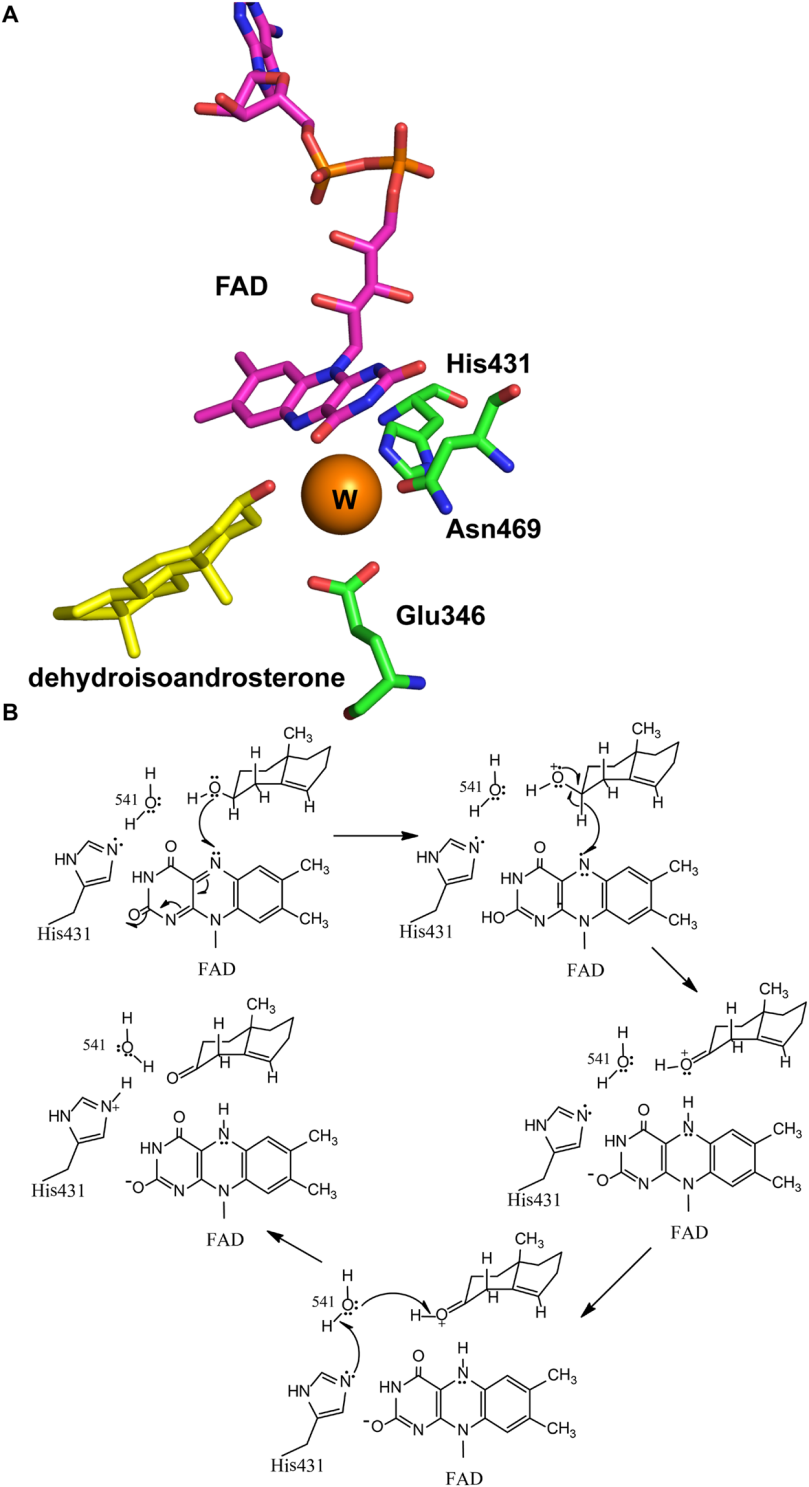
Catalytic residues at active site (**A**) and proposed catalytic mechanism (**B**)^24^.

The active site is located under the isoalloxazine of FAD for the noncovalent form in ChO family enzymes^7^. Three residues, H431, E346 and N469, form a catalytic triad (Fig. 3). Residue H431 acts as the general base catalyst for abstraction of a proton from the C3-OH of the steroid substrate during oxidation and, subsequently, as the general acid for stabilization of the dienolic intermediate of isomerization^23^. E346 is positioned in close proximity to the C4 β-proton of the steroid. E346 acts as the base for both catalytic reactions to transfer a proton and the isomerization step^13^. N469 affects the oxidative activity and modulates the redox potential of the flavin through interaction between its side chain and the ring of FAD^26^. A single water molecule at the active site forms a network of hydrogen bonds between the catalytic residues H431, E346, N469 and the C3-OH of the substrate. This active water molecular acts as the keystone for the active site^27,28^.

### Enzyme characteristics of PsChO

**Figure 4:**
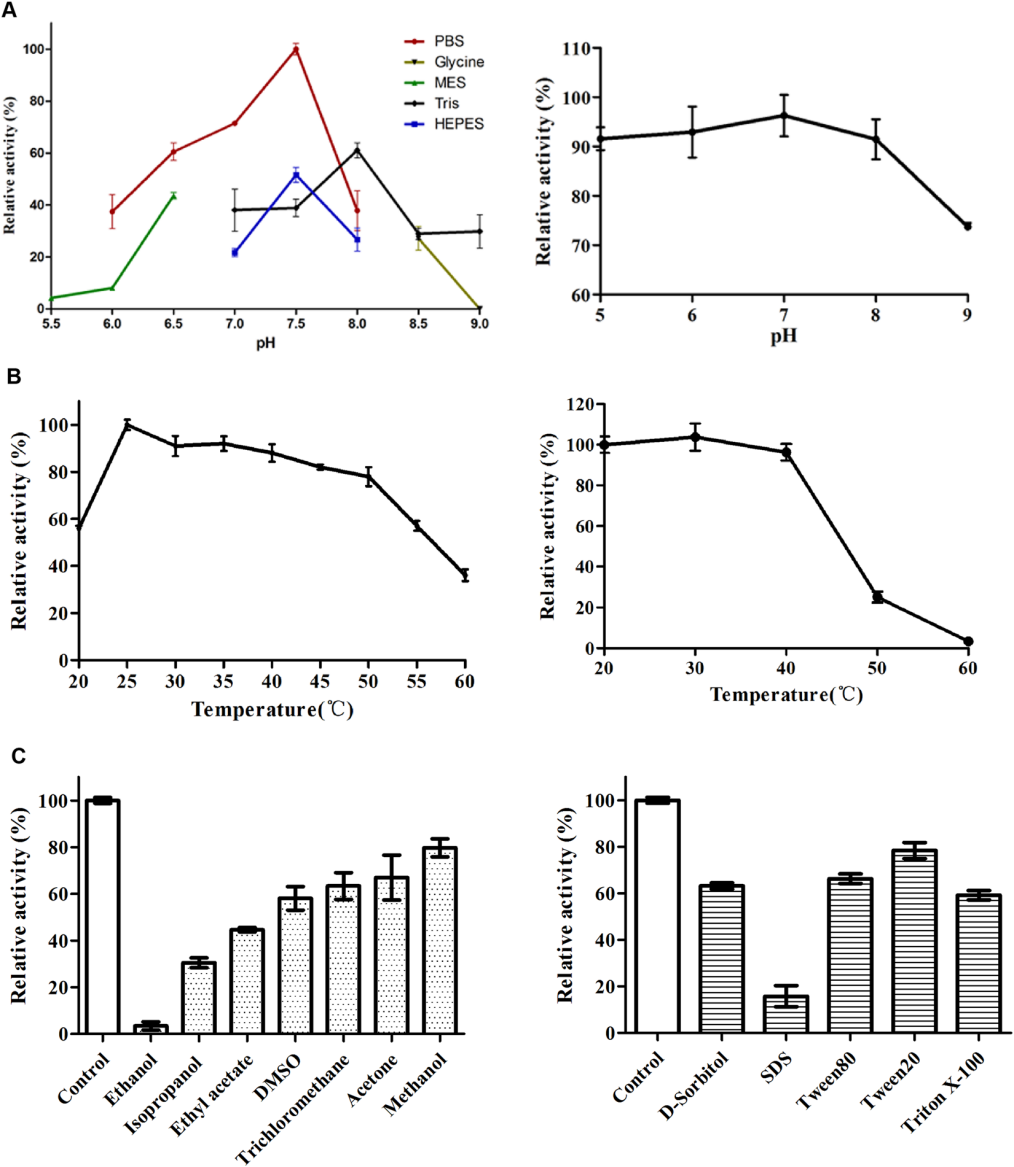
Effect of pH, temperature, organic solvents and detergents on activity of PsChO. (**A**) pH dependence of PsChO (Left): Activity was measured in the reaction mixtures adjusted to various pHs with 25 mM MES, PBS, HEPES, Tris-HCl, and glycine-NaOH buffers, respectively. Analysis of pH stability of PsChO (Right): The PsChO was pre-incubated at different pH values for 1 h at 4 °C, and the residual activity was determined in the standard assay conditions. (**B**) Temperature dependence of PsChO (Left): Activity was measured at various temperatures in the standard assay conditions. Thermostability analysis of PsChO (Right): The PsChO was pre-incubated at various temperatures for 30 min, and the residual activity was determined in the standard assay condition. (**C**) Organic solvents and detergents were added to the solution at 4 °C for 1 h. The relative residual activity shows the activity as compared to that observed in a control PsChO solution not exposed to the organic solvent/detergents. The activities of control PsChO are represented as 100 and the error bars are standard deviations (n = 3).

The catalytic activity of ChO is highly dependent on the buffer composition, pH, temperature and surfactant used to solubilize the substrate; a comparison and discussion of these parameters were performed. The purified WT PsChO was active at pH 5.5–9.0 and maximal activity was observed in PBS buffer pH 7.5. The PsChO exhibited good pH stability over the pH range of 5.0–9.0 and more than 75% of the maximal activity was retained over this range (Fig. 4A). The optimal temperature for PsChO activity was 25 °C. The activity began to decrease when the temperature was >40 °C, and there was only 25% residual activity after pre-incubation at 50 °C for 30 minutes (Fig. 4B). Cholesterol oxidase catalyzed substrates in organic solvents and detergents using hydrophobic interactions due to the negligible solubility of cholestane skeleton substrates in buffers. The catalytic activity of PsChO in various micelles varied, but all micelle solutions reduced enzyme activity (Fig. 4C).

**Figure 5:**
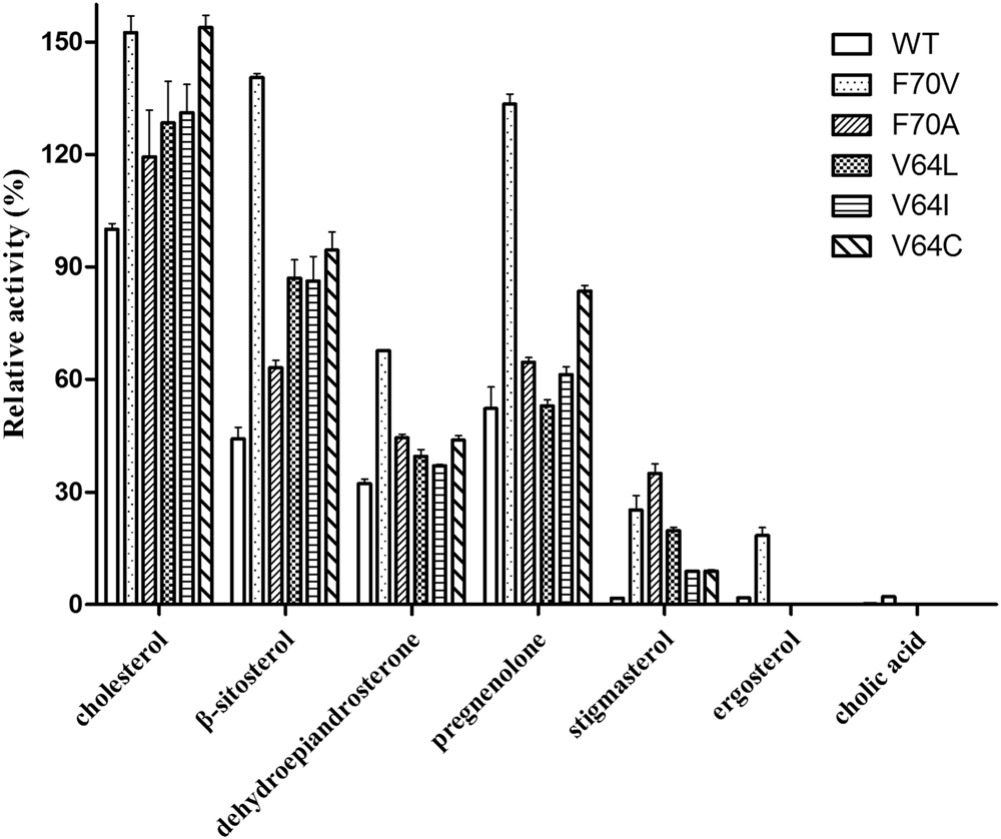
The relative activity of PsChO mutants towards seven substrates. The activity of WT PsChO toward cholesterol substrate is represented as 100 and the error bars are standard deviations (n = 3).

PsChO exhibited a broad range of selectivity toward various cholestane skeleton substrates, especially the 3β-hydroxysteroids (Fig. 5). However, PsChO showed almost no activity toward 3α-hydroxysteroid (cholic acid), because His431 might not abstract a proton from the 3α-O of cholic acid (Fig. S4). The highest enzyme activity was observed using cholesterol as the substrate (12.7 U mg^−1^), and the products of cholestane skeleton substrates, such as cholest-4-en-3-one, β-sitostenone, stigmastadienone, progesterone, ergosta-4,7,22-trien-3-one and androstenedione were characterized by GC-MS (Fig. S5).

### Structure-based rational design of PsChO

To identify the role of specific amino acids in substrate selectivity and structure-function relationships, a single point mutant library containing only 39 single mutants at 17 residues was constructed and evaluated (Figs 5 and S6). Mutation of residues V46, P63, M109, M205, L360, P331, F344, and Y430 reduced activity, indicating that these residues play a crucial role in substrate recognition. Interestingly, P63 and P432 mutants were inactive toward cholesterol. Mutation of V64 and F70 improved the catalytic activity toward cholesterol (Fig. S6). Therefore, these two sites were selected as targets for rational design.

Proteins are dynamic and undergo conformational changes to bind ligands, which makes protein modification a major challenge in structure-based rational design. In order to increase enzyme activity, we performed rational design beginning with site-directed mutagenesis guided by the structural homology model of PsChO. The mutants V64L, V64I, V64C, F70V and F70A showed improved activities toward cholestane skeleton substrates. These mutants also showed higher *k*_cat_/*K*_m_ values toward cholesterol when compared with the *k*_cat_/*K*_m_ value of the WT protein (Table S2). The F70L mutant showed almost the same level of activity as the WT, which suggests that the leucine side chain did not effectively enlarge the binding pocket to reduce the steric hindrance with the substrate. Surprisingly, mutants V64C and F70V showed 50% higher activities toward cholesterol when compared with that of the WT enzyme. Furthermore, these two mutants, together with V64L/I and F70A also improved the activity toward β-sitosterol, dehydroepiandrosterone, pregnenolone and stigmasterol (Figs 5, S7 and Table 1). Unexpectedly, the F70 mutants improved catalytic activity >10-fold toward ergosterol and stigmasterol when compared with the results of the WT enzyme (Fig. S8).

**Table 1:** Kinetic parameters of PsChO WT and variants toward five substrates.

		WT	F70V	V64C
cholesterol	*K*_m_ (μM)	204.85 ± 3.14	154.83 ± 3.31	178.31 ± 1.38
	*k*_cat_ (s^−1^)	12.29 ± 0.32	15.48 ± 0.30	23.02 ± 0.29
	*k*_cat_/*K*_m_ (μM^−1^s^−1^)	0.06 ± 0.0003	0.10 ± 0.0004	0.13 ± 0.0005
β-sitosterol	*K*_m_ (μM)	328.61 ± 3.60	178.42 ± 1.61	220.76 ± 3.14
	*k*_cat_ (s^−1^)	9.86 ± 0.35	16.06 ± 0.33	13.25 ± 0.36
	*k*_cat_/*K*_m_ (μM^−1^s^−1^)	0.03 ± 0.0005	0.09 ± 0.0008	0.06 ± 0.0007
dehydroepiandrosterone	*K*_m_ (μM)	341.21 ± 3.79	273.52 ± 2.13	318.81 ± 3.25
	*k*_cat_ (s^−1^)	6.82 ± 0.14	13.68 ± 0.20	9.56 ± 0.34
	*k*_cat_/*K*_m_ (μM^−1^s^−1^)	0.02 ± 0.0004	0.05 ± 0.0006	0.03 ± 0.0005
pregnenolone	*K*_m_ (μM)	294.55 ± 2.97	190.61 ± 3.30	228.42 ± 3.24
	*k*_cat_ (s^−1^)	11.78 ± 0.34	15.25 ± 0.22	13.71 ± 0.31
	*k*_cat_/*K*_m_ (μM^−1^s^−1^)	0.04 ± 0.0005	0.08 ± 0.0004	0.06 ± 0.0006
stigmasterol	*K*_m_ (μM)	789.61 ± 4.71	384.12 ± 3.84	528.84 ± 3.59
	*k*_cat_ (s^−1^)	1.58 ± 0.07	7.68 ± 0.27	4.23 ± 0.19
	*k*_cat_/*K*_m_ (μM^−1^s^−1^)	0.002 ± 0.0001	0.02 ± 0.0002	0.008 ± 0.0002

The Michaelis–Menten constant (*K*_m_) and the catalytic rate constant (*k*_cat_) of PsChO toward cholesterol were determined to investigate the substrate selectivity. The *k*_cat_/*K*_m_ of WT for cholesterol (0.06 μM^−1^ s^−1^) was higher than other substrates, which implied that PsChO possessed strong catalytic activity towards the C17 hydrophobic substrate with a long side chain when compared with the activity toward smaller substrates (i.e. dehydroepiandrosterone and pregnenolone). However, the F70V mutant had a more than two-fold increased *k*_cat_/*K*_m_ toward cholesterol, β-sitosterol, dehydroepiandrosterone and pregnenolone when compared with the results for the WT enzyme. A similar increased in activity was also observed for the V64C mutant.

### Molecular dynamic simulations

**Figure 6:**
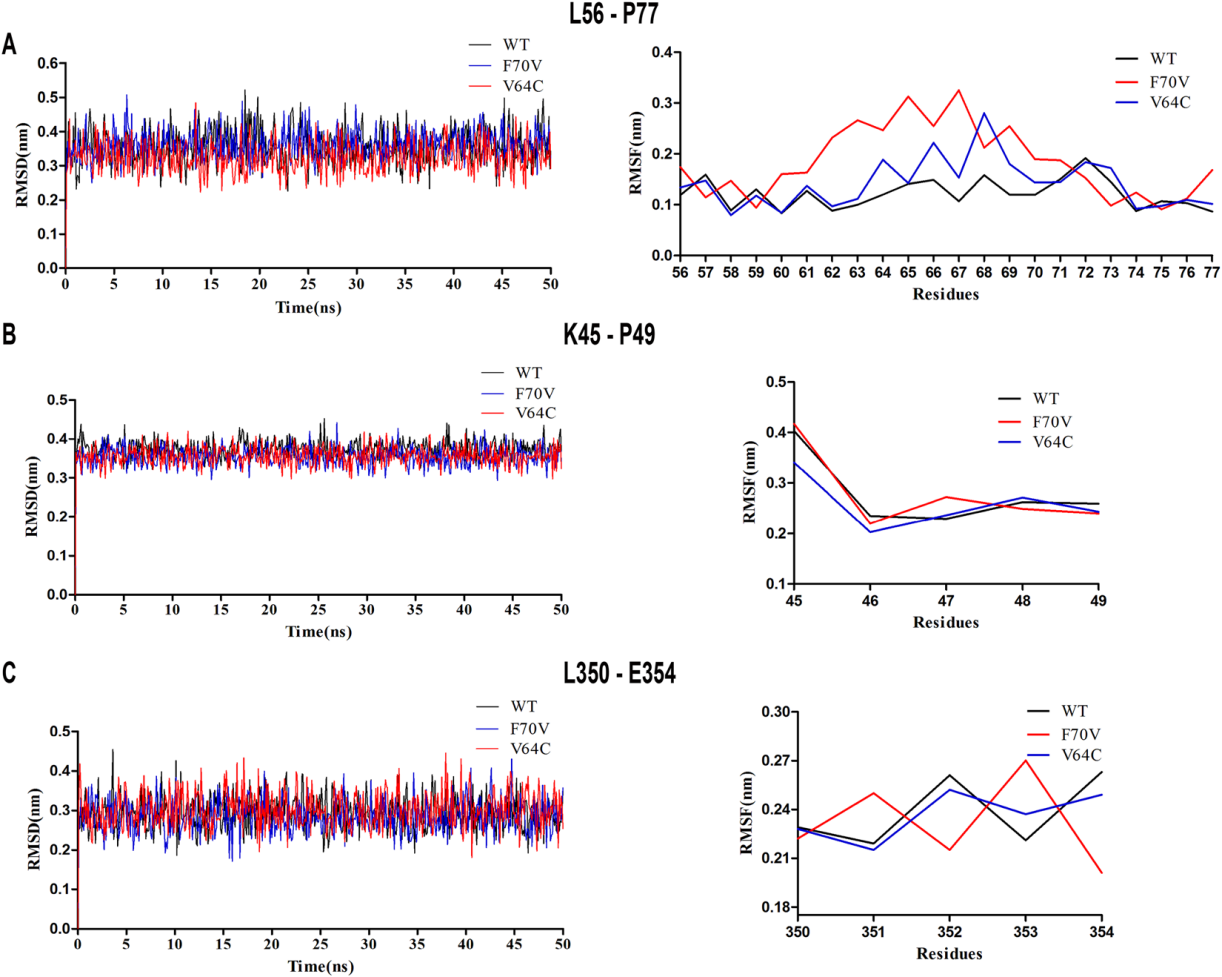
The RMSD and RMSF of cholesterol and residues around the binding sites over 50-ns simulations with respect to their initial position for the protein in the complexes systems. The black, blue and red lines represent proteins of wild-type, F70V and V64C mutants, respectively.

Molecular dynamic simulations were performed to investigate the binding free energy of PsChO WT and mutants with cholesterol. The results showed that the binding energies of PsChO mutants with cholesterol were increased compared with that of WT (>−104 kcal/mol vs −96.83 kcal/mol) (Table 2). Notably, the V64C mutant contributed the highest affinity toward cholesterol. Thus, the PsChO mutants exhibited higher substrate affinity than the WT enzymes. To determine the stability of PsChO/mutants–cholesterol complexes, RMSD and root mean square fluctuations (RMSF) of the whole system over 50-ns are shown in Fig. 6. Some residues in loops of K45-P49, A62-F70 and T352-I354, which are part of the substrate binding sites showed a greater degree of flexibility with RMSFs of >0.2 nm when compared with that of the WT enzyme, indicating that these residues are more flexible as a result of binding to ligands.

**Table 2:** Binding energy of PsChO-cholesterol for WT and various mutants (kcal/mol).

	ΔG_MM_^a^	ΔG_Polar_^b^	ΔG_Apolar_^c^	ΔG_binding_^d^
WT	−90.82	5.09	−11.07	−96.83
V64C	−104.44	4.53	−12.20	−112.10
V64L	−97.60	2.27	−11.59	−106.93
V64I	−95.56	2.28	−11.54	−104.81
F70A	−98.07	3.38	−11.12	−105.83
F70V	−98.11	2.50	−12.19	−107.80

^a^potential energy in vacuum; ^b^polar-solvation energy; ^c^non-polar solvation energy; ^d^ΔG_binding_ = ΔG_MM_ + ΔG_Polar_ + ΔG_Apolar_.

### Structural comparison of PsChO wild type and mutants

**Figure 7:**
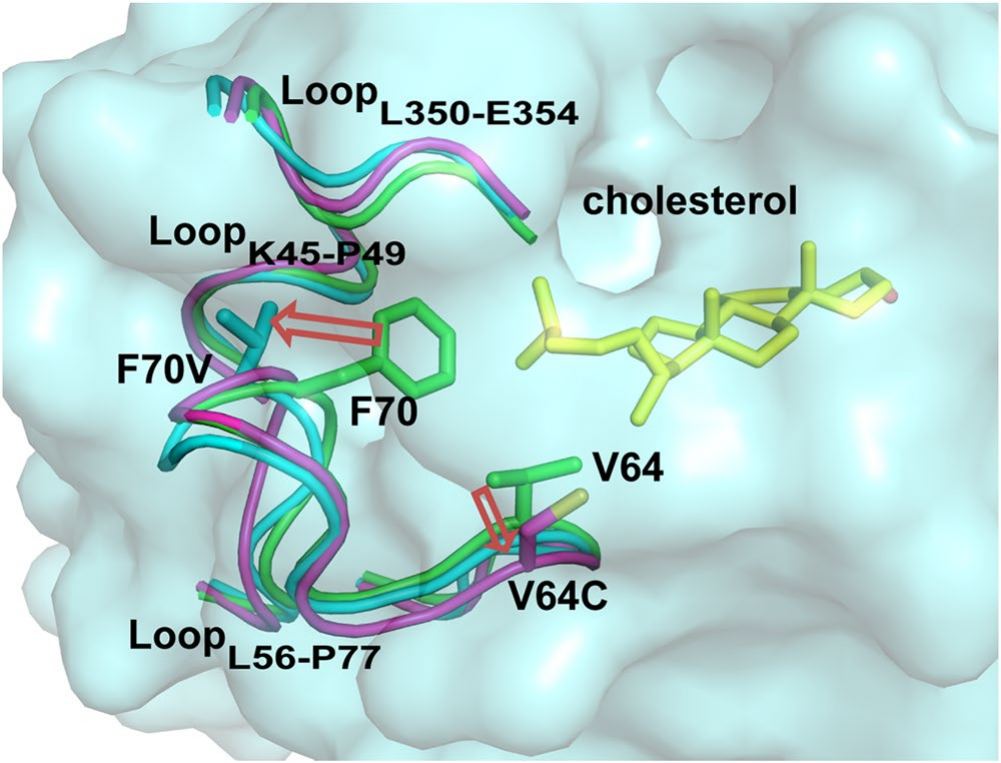
Ribbon representation of the MD-derived structures of PsChO WT and mutants with cholesterol bound. The PsChO WT, F70V and V64C mutants are shown in green, cyan and magenta, respectively. Cholesterol is shown as a yellow stick model.

A structural comparison between PsChO and family members reveals a structural change to residues L56-P77 (Figs 2C, S2 and S3), where the side chains of V64 and F70 located in the lid loop project into the active-site cavity, regulating the entrance on protein surface (Fig. 2C). We compared the equilibrated 3D structures from 50 ns of MD trajectory for WT PsChO and mutants to obtain further insights into protein conformational changes associated with substrate binding. The features of the structural and functional analyses are supported by the MD simulations in which V64 and F70 within the loop have moved upon substrate binding, resulting in an enlargement of the steroid-binding cavity. The movement of the lid loop (L56-P77) including the side-chain of V64 and F70 facilitates the accommodation of the hydrophobic tail moiety of long C17 side-chain substrates (Figs 6 and 7 and S9). These results also suggest that the mutations cause movement of two other loops (K45-P49 and L350-E354), which are located near the substrate cholesterol and interacted with the tail moiety of cholesterol. Therefore, F70V and V64C appear to relieve the steric interference with substrates by forming a sufficiently large space to accommodate larger substrates in the active site.

We have characterized PsChO, showing that this enzyme exhibits pH stability over the range of 5.0–9.0 and is thermostable to 40 °C. PsChO catalyzes 3β-hydroxysteroids, i.e. cholesterol, β-sitosterol and pregnenolone. Residues related to substrate recognition around the active site were investigated and mutants of V64 and F70 improved the substrate selectivity and activity. Consistent with structural data, the MD simulations showed that PsChO mutants increased the binding free energy when compared with that of the WT. Furthermore, the lid loop of L56-P77, including V64 and F70, regulated substrate entry. The substrate selectivity of PsChO was significantly improved toward various cholestane skeleton substrates based on rational design and protein engineering technologies.

## Materials and Methods

### Cloning, expression and purification

The *E. coli* BL21(DE3) cells harboring pET28a(+) with the wild-type PsChO gene (GenBank No. CP009896.1; Protein ID: WP_052138420.1) were grown at 37 °C in lysogeny broth medium containing 40 μg/mL kanamycin. Overexpression was induced by adding 0.1 mM isopropyl-β-D-thiogalactopyranoside (IPTG) when OD_600_ reached 0.6–0.8. The culture was then further incubated at 16 °C overnight. After harvesting, the cells were disrupted by sonication in the resuspending buffer [20 mM Tris-HCl (pH 7.4), 20 mM imidazole, and 0.5 M NaCl], and the cell debris was removed by centrifugation. PsChO was trapped on Ni-NTA Superflow resin (QIAGEN). After washing, the His_6_-tagged protein was eluted with resuspension buffer containing 400 mM imidazole. The solution containing purified PsChO was dialyzed against PBS buffer for activity assay. The PsChO concentration was measured by the method of BCA.

### Activity assay

The activity of cholesterol oxidase was measured using the following protocol^10^. The purified enzyme was added to 3 mL solution A containing 25 mM phosphate buffer (pH 7.5), 1 mM 4-aminoantipyrine, 6 mM phenol, 7000 U L^−1^ peroxidase and solution B containing 150 μL isopropanol with 0.83% (w/v) cholesterol and 4.26% (v/v) Triton X-100. The reaction was proceeded for 5 min and was terminated by placing the reaction mixture in boiling water. The concentration of H_2_O_2_ was calculated by the increase in the OD_500_ value. One units of enzyme is defined as the amount of enzyme to catalyze 1 μM cholesterol in one minute.

### Characterization of PsChO

The optimal pH of the purified enzyme was determined in the following buffers: 25 mM MES buffer (pH 5.5–6.5), 25 mM PBS buffer (pH 6.0–8.0), 25 mM HEPES buffer (pH 7.0–8.0), 25 mM Tris-HCl buffer (pH 7.0–9.0) and 25 mM glycine-NaOH buffer (pH 8.5–9.0) at 25 °C for 5 min. The effect of pH on PsChO stability was determined by incubating the purified enzymes under various pH conditions (pH 5.0–9.0) for 1 h at 4 °C. The buffers (25 mM each) used were: pH 5.0–6.0, MES; pH 7.0–8.0, PBS; pH 9.0, Tris-HCl. Residual enzyme activity was then measured. The optimal temperature was determined by incubating PsChO at different temperatures ranging from 20 to 60 °C. The thermostability was determined by measuring the residual activity after the incubation of the enzyme at different temperatures (20–70 °C) for 30 min in PBS buffer (pH 7.5). The effects of organic solvents (33% v/v, ethanol, isopropanol, ethyl acetate, DMSO, trichloromethane, acetone and methanol) and detergents (0.5% w/v, SDS, D-sorbitol; 0.5% v/v Tween80, Tween20 and Triton X-100) to the activity of the protein was determined by measuring the residual activity after incubation of the enzyme at 4 °C for 1 h in the various organic solvents and detergents. The activity was then measured under standard reaction conditions.

### GC-MS analysis of products of the cholestane skeleton substrates

The products of cholestane skeleton substrates, such as cholest-4-en-3-one, β-sitostenone, stigmastadienone, progesterone, ergosta-4,7,22-trien-3-one and androstenedione were resuspended in 20 μL n-hexane and a 1-μL aliquot analyzed by GC-MS (VARIAN 4000 GC/MS) using an ultraviolet detector (Agilent 1260 Infinity, USA) at 240 nm and a HP-5 ms (30 m × 0.25 mm × 0.25 mm, Agilent Technologies) column in electron ionization (70 eV) mode. Initial oven temperature was 150 °C, held for 3 min, then increased to 300 °C at the rate of 5 °C/min and then held for 10 min. Helium was used as a carrier gas at a flow rate of 1 ml/min. Temperatures of injection port, interface and ion source were 280 °C, 250 °C and 200 °C, respectively.

### Structure modeling of the PsChO

The three-dimensional (3D) homology model of PsChO was generated using Modeller 9.9^29^. The crystal structure of cholesterol oxidase from *Brevibacterium sterolicum* (PDB ID: 1COY, 1.8 Å), which has 66% sequence identity to the target protein PsChO, was chosen as the template. The align2d command was used to automatically generate a sequence alignment between the template and PsChO. Subsequently, homology modeling was performed by the automodel command. Thereafter, each model was first optimized by the variable target function method with conjugate gradients. Simulated annealing MD simulations were used to refine the structure. Finally, the best model was chosen based on the values of the Modeller objective function and the DOPE assessment scores. The PyMol molecular Graphics System (http://www.pymol.org)^30^ was used to visualize and analyze the generated model structure.

### Molecular dynamics simulations

The initial structure of cholesterol was built by Chemoffice software. The GROMOS96 53a6 force-field parameters of cholesterol were sourced from the Automated Topology Builder and Repository 2.0 webserver (https://atb.uq.edu.au/)^31^. Subsequently, the atomic charges and charge groups of brazilin were corrected to achieve better agreement with the GROMOS96 53A6 force field parameter set^32^.

The cholesterol and the target protein PsChO complex were initially placed into a square box with a size of 7 nm. Water molecules were then added into the box and three water molecules were replaced by the same number of negative Cl^−^ ions. The simple point charge (SPC) model was used to describe water. We first performed 1000 energy minimization steps to relax the simulation system, after which the relaxed system was equilibrated for 40 ps by successively using an isochoric-isothermal ensemble and an isothermal-isobaric ensemble, via the Berendsen weak coupling method^33^. Finally, the product MD simulations of 10 ns under different initial conditions were carried out by assigning different initial velocities to each atom of the simulation system. All of the MD simulations were performed close to physiological temperature (i.e., 300 K) and a pressure of 1 bar.

We performed all-atom MD simulations using the GROMACS 5.1.1 package^34^ together with the GROMOS96 53A6 force field. Newton’s classical equations of motion were integrated using the Verlet Leapfrog algorithm with a 2 fs time step^35^. All short-range non-bonded interactions were cut off at 1.4 nm, with dispersion correction applied to energy and pressure terms to account for the truncation of van der Waals interactions. Long-range electrostatic interactions were calculated with the smooth particle mesh Ewald method^36^ via cubic-spline interpolation with a Fourier grid spacing of approximately 0.12 nm. The neighbor list was updated every five simulation steps. All bond lengths were constrained using the LINCS algorithm^37^ with a relative geometric tolerance of 10^−4^. Initial velocities were assigned according to a Maxwell distribution. The atomic coordinates were saved every 50 ps for subsequent analyses. The snapshots were made using PyMol version 0.99rc6^30^. The free energies between cholesterol and the target protein were calculated using g_mmpbsa software^38^.

### Site-directed mutagenesis

Site-directed mutagenesis of PsChO was performed by PCR with the QuikChange method on a template consisting of the DNA encoding PsChO inserted into pET28a(+) vector. The primers for mutants are summarized in Table S1. The mutations were confirmed by DNA sequencing. PsChO mutants were expressed and purified according to the method described for wild-type PsChO.

## Electronic supplementary material

supporting information
