# Supplementary material for: Rational design of cholesterol oxidase for efficient bioresolution of cholestane skeleton substrates
Source: Sci Rep. 2017 Nov 27;7:16375. doi: 10.1038/s41598-017-16768-6 (PMC5703901; doi:10.1038/s41598-017-16768-6)
Supplement: Supplementary file 1 — supporting information [file 41598_2017_16768_MOESM1_ESM.pdf]

Supporting Information for

Rational design of cholesterol oxidase for efficient bioresolution of cholestane skeleton substrates

Hui-Min Qin[1,2,3,4], Zhangliang Zhu[4], Zheng Ma[4], Panpan Xu[4], Qianqian Guo[4], Songtao Li[4], Jian-Wen Wang[4], Shuhong Mao[1,2,3,4], Fufeng Liu[1,2,3,4,5]*, Fuping Lu[1,2,3,4,5]*

[1]*State Key Laboratory of Food Nutrition and Safety, P. R. China*

[2]*Key Laboratory of Industrial Fermentation Microbiology, Ministry of Education, P. R. China*

[3]*Tianjin Key Laboratory of Industrial Microbiology, P. R. China*

[4]*College of Biotechnology, Tianjin University of Science and Technology, P. R. China*

[5]*National Engineering Laboratory for Industrial Enzymes, Tianjin 300457, P. R. China*

*Corresponding authors: Fufeng Liu; Fuping Lu

Mailing address: No 29, 13th Avenue, Tianjin Economic and Developmental Area (TEDA), Tianjin， 300457, China

Tel: +86-22-60601958. Fax: +86-22-60602298

E-mail: fufengliu@tust.edu.cn; lfp@tust.edu.cn

Table S1. Primers used for the construction of recombinant PsChO.

Primer name	Sequence of primer
WT_F	GGAATTC**CATATG**GTGCTCGTCATCGGCAC
WT_R	CCG**GAATTC**CTAGATGTCGTTGGCCAGG
V46F_F	ATCTTCGCGAAG**GCC**ACCTCGCCCGAC
V46F_R	GTCGGGCGAGGT**GGC**CTTCGCGAAGAT
V46G_F	GATCTTCGCGAAG**GGC**ACCTCGCCCGAC
V46G_R	GTCGGGCGAGGT**GCC**CTTCGCGAAGATC
V46H_F	CAAGATCTTCGCGAAG**CAC**ACCTCGCCCGACAAG
V46H_R	CTTGTCGGGCGAGGT**GTG**CTTCGCGAAGATCTTG
V46M_F	CAAGATCTTCGCGAAG**ATG**ACCTCGCCCGACAAGC
V46M_R	GCTTGTCGGGCGAGGT**CAT**CTTCGCGAAGATCTTG
A62Q_F	CGCACCAAGACCAAG**CAG**CCGGTGAGCAACTTC
A62Q_R	GAAGTTGCT**CAC**CGGC**TGC**TTGGTCTTGGTGCG
P63D_F	GCACCAAGACCAAGGCG**GAT**GTGAGCAACTTCTTCGG
P63D_R	CCGAAGAAGTTGCTCAC**ATC**CGCCTTGGTCTTGGTGC
P63S_F	CCAAGACCAAGGCG**TCG**GTGAGCAACTTC
P63S_R	GAAGTTGCTCAC**CGA**CGCCTTGGTCTTGG
P63T_F	CCAAGACCAAGGCG**ACG**GTGAGCAACTTC
P63T_R	GAAGTTGCTCAC**CGT**CGCCTTGGTCTTGG
V64A_F	ACCAAGGCGCCG**GCG**AGCAACTTCTTCG
V64A_R	CGAAGAAGTTGCT**CGC**CGGCGCCTTGGT
V64C _F	CCAAGACCAAGGCGCCG**TGC**AGCAACTTCTTCGGCTT
V64C_R	AAGCCGAAGAAGTTGCT**GCA**CGGCGCCTTGGTCTTGG
V64F _F	CAAGACCAAGGCGCCG**TTC**AGCAACTTCTTCGGC
V64F_R	GCCGAAGAAGTTGCT**GAA**CGGCGCCTTGGTCTTG
V64H _F	CCAAGACCAAGGCGCCG**CAT**AGCAACTTCTTCGGC
V64H_R	GCCGAAGAAGTTGCT**ATG**CGGCGCCTTGGTCTTGG
V64I _F	CAAGACCAAGGCGCCG**ATA**AGCAACTTCTTCGGCT
V64I _R	AGCCGAAGAAGTTGCT**TAT**CGGCGCCTTGGTCTTG
V64L_F	AAGACCAAGGCGCCG**TTG**AGCAACTTCTTCG
V64L _R	CGAAGAAGTTGCT**CAA**CGGCGCCTTGGTCTT
V64M _F	CAAGACCAAGGCGCCG**ATG**AGCAACTTCTTCG
V64M _R	CGAAGAAGTTGCT**CAT**CGGCGCCTTGGTCTTG
V64T _F	CAAGACCAAGGCGCCG**ACG**AGCAACTTCTTCGGC
V64T_R	GCCGAAGAAGTTGCT**CGT**CGGCGCCTTGGTCTTG
F67L_F	AGGGAAGCCGAA**TAA**GTTACTCACCGGTGCTTTGG
F67L_R	CCAAAGCACCGGTGAGTAAC**TTA**TTCGGCTTCCCT
F67A_F	ATAGGGAAGCCGAA**AGC**GTTACTCACCGGTGCTTTGGTCT
F67A_R	AGACCAAAGCACCGGTGAGTAAC**GCT**TTCGGCTTCCCTAT
F70A_F	AGCAACTTCTTCGGC**GCC**CCCATCGACGCCAC
F70A _R	GTGGCGTCGATGGG**GGC**GCCGAAGAAGTTGCT
F70V _F	GCAACTTCTTCGGC**GTC**CCCATCGACGCC
F70V _R	GGCGTCGATGGG**GAC**GCCGAAGAAGTTGC

F70L _F	GAGCAACTTCTTCGGC**TTA**CCCATCGACGCC
F70L _R	GGCGTCGATGGG**TAA**GCCGAAGAAGTTGCTC
M109G _F	CGTCAACGGCGGC**GGG**GCAGTCACGCCG
M109G _R	CGGCGTGACTGC**CCC**GCCGCCGTTGACG
M109V _F	CTCGTCAACGGCGGC**GTG**GCAGTCACGC
M109V _R	GCGTGACTGC**CAC**GCCGCCGTTGACGAG
M205H _F	CGTGGTCGTTGCCGTA**ATG**GACCTGGCCGTCGAGC
M205H _R	GCTCGACGGCCAGGTC**CAT**TACGGCAACGACCACG
M312C_F	GTGGTTTGCACGGCC**GCA**GCACACGTTACCATTATCGCC
M312C_R	GGCGATAATGGTAACGTGTGC**TGC**GGCCGTGCAAACCAC
P331A_F	CAGTCGACGAT**CGC**CTGCTCGGGCA
P331A_R	TGCCCGAGCAG**GCG**ATCGTCGACTG
F344C_F	CCTCGGC**GCA**GGCACCACCGGCGT
F344C_R	ACGCCGGTGGTGCC**TGC**GCCGAGG
F344D_F	GCGACCTCGGC**GTC**GGCACCACCGGC
F344D_R	GCCGGTGGTGCC**GAC**GCCGAGGTCGC
E346D_F	AGCGGGGCGAC**ATC**GGCGAAGGC
E346D_R	GCCTTCGCC**GAT**GTCGCCCCGCT
E346T_F	CAGCGGGGCGAC**CGT**GGCGAAGGCACCA
E346T_R	TGGTGCCTTCGCC**ACG**GTCGCCCCGCTG
L350A_F	AATGCCGGTCGG**CGC**CGGTGCCACTTCG
L350A_R	CGAAGTGGCACCG**GCG**CCGACCGGCATT
L360A_F	GAGACCTACGCCTCG**GCC**TACCTGTCGATCAC
L360A_R	GTGATCGACAGGTA**GGC**CGAGGCGTAGGTCTC
L360G_F	GTGATCGACAGGTA**GCC**CGAGGCGTAGGTCTC
L360G_R	GAGACCTACGCCTCG**GGC**TACCTGTCGATCAC
L428F_F	GGGTGGTAGGT**GAA**GCCGTCCTGCCAG
L428F_R	CTGGCAGGACGGC**TTC**ACCTACCACCC
Y430A_F	GCCCAGCGGGTG**GGC**GGTGAGGCCGTCC
Y430A_R	GGACGGCCTCACC**GCC**CACCCGCTGGGC
Y430F_F	CCAGCGGGTG**GAA**GGTGAGGCCGTC
Y430F_R	GACGGCCTCACC**TTC**CACCCGCTGG
Y430V_F	GGACGGCCTCACC**GTC**CACCCGCTGGGC
Y430V_R	GCCCAGCGGGTG**GAC**GGTGAGGCCGTCC
P432D_F	CACCGCACCGCCCAG**ATC**GTGGTAGGTGAGGCC
P432D_R	GGCCTCACCTACCAC**GAT**CTGGGCGGTGCGGTG
P432K_F	CGCACCGCCCAG**CTT**GTGGTAGGTGAGG
P432K_R	CCTCACCTACCAC**AAG**CTGGGCGGTGCG

Table S2 Kinetic parameters of PsChO wild-type and variants towards cholesterol.

	K_m (μM)	k_{cat} (s^{-1})	k_{cat}/K_m ($\mu M^{-1}\ s^{-1}$)
WT	204.85 ±3.14	12.29 ±0.32	0.06 ±0.0003
F70A	178.67 ±3.59	14.67 ±0.43	0.08 ±0.0006
F70V	154.83 ±3.31	15.48 ±0.30	0.10 ±0.0004
V64I	184.91 ±4.08	19.14 ±0.35	0.10 ±0.0007
V64L	183.43 ±3.30	18.84 ±0.25	0.10 ±0.0008
V64C	178.31 ±1.38	23.02 ±0.29	0.13 ±0.0005

All parameters are the average of three independent determinations, with the range between values being always less than ±5%.

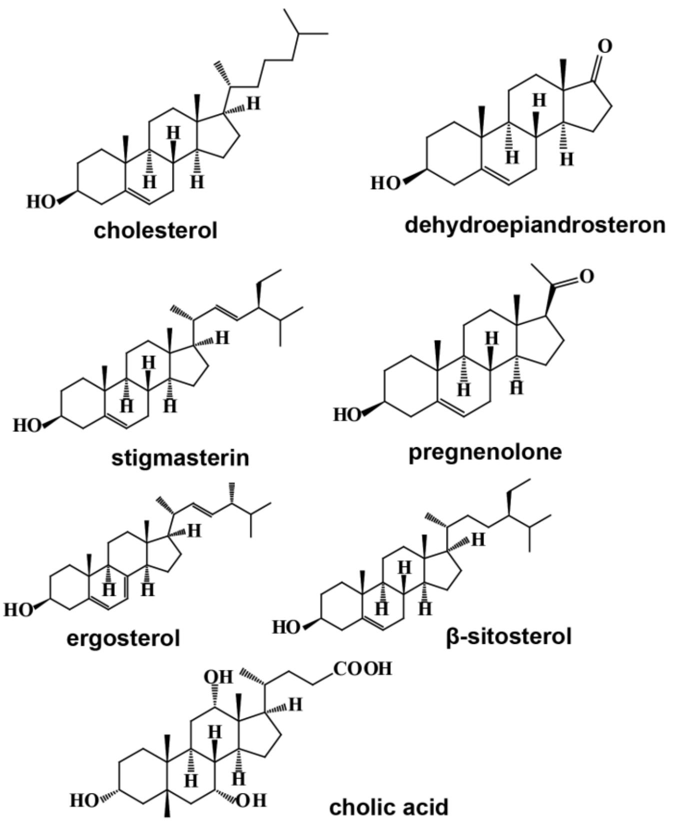

Fig. S1 Substrates used for PsChO catalysis.

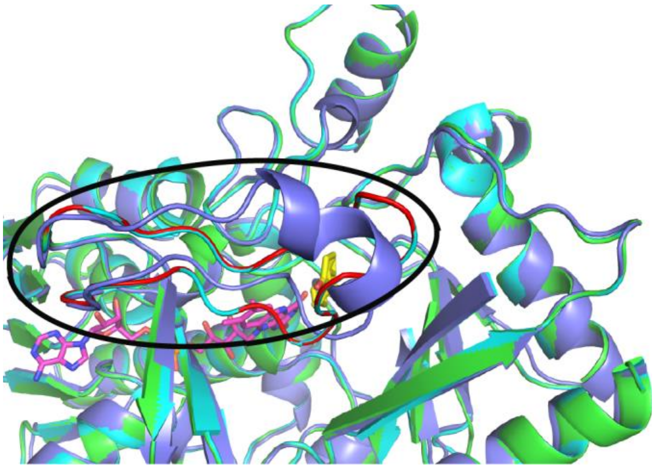

Fig. S2 Superposition of the flexible lids of PsChO (green) with other family members (PDB ID: 1B4V, blue and PDB ID: 1COY, cyan). The largest conformational differences among the three structures are circled.

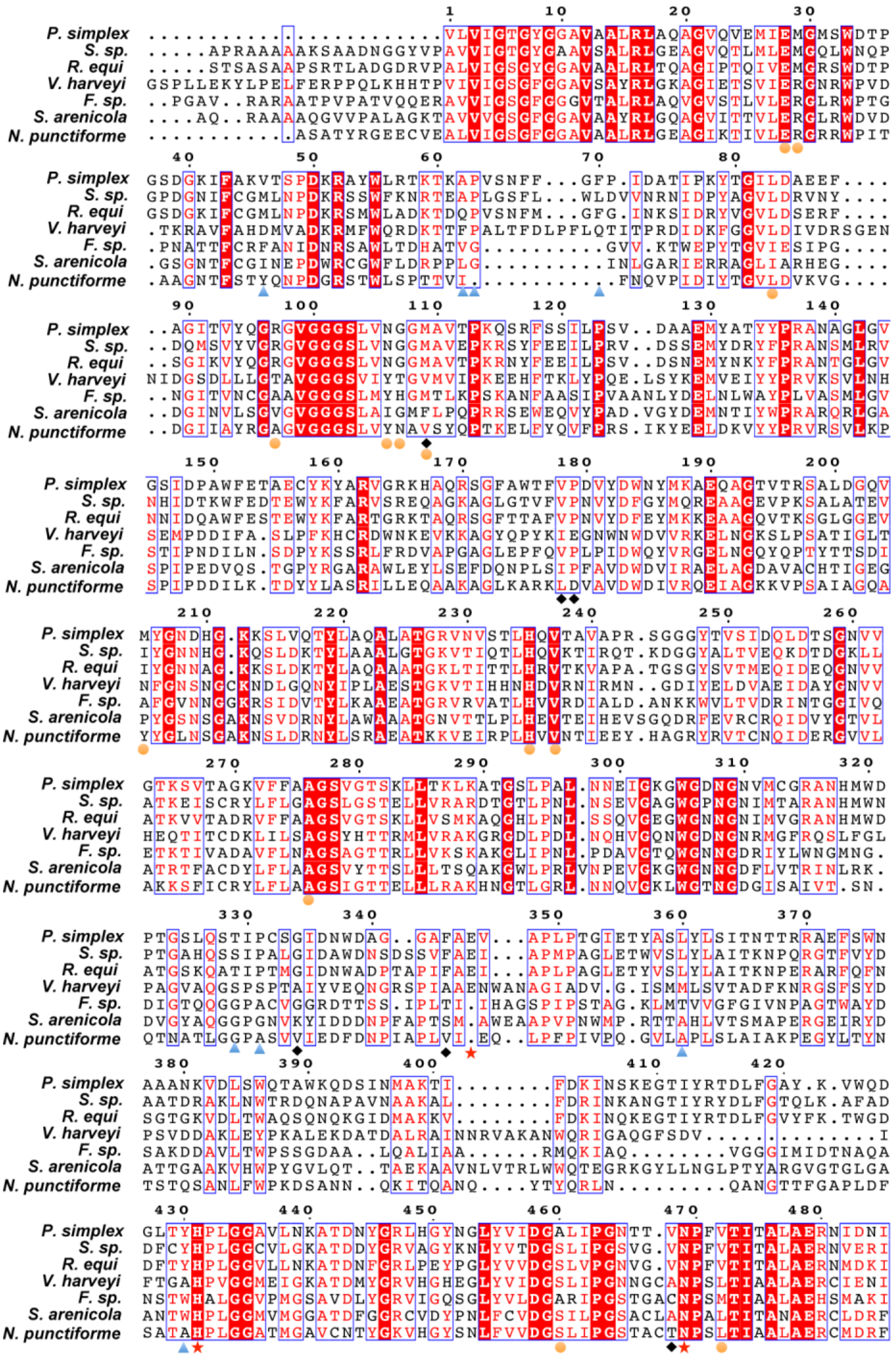

Fig. S3 Amino acid sequence alignments of cholesterol oxidases from different strains. The catalytic residues were marked as red asterisk. FAD binding residues were marked as yellow circle. The substrate-binding residues were marked as blue triangle. The residues in narrow-gated oxygen channel were marked as black diamond.

P. simplex: *Pimelobacter simplex*, CP009896.1; *S.* sp.: *Streptomyces* sp., AAA26719; *R. equi*: *Rhodococcus equi*, CAC44897; *V. harveyi*: *Vibrio harveyi* HY01, ZP_01986092; *F.* sp.: *Frankia* sp. EAN1pec, WP_020461426; *S. arenicola*: *Salinispora arenicola* CNS-205, WP_012182946; *N. punctiforme*: *Nostoc punctiforme*, ACC84788.

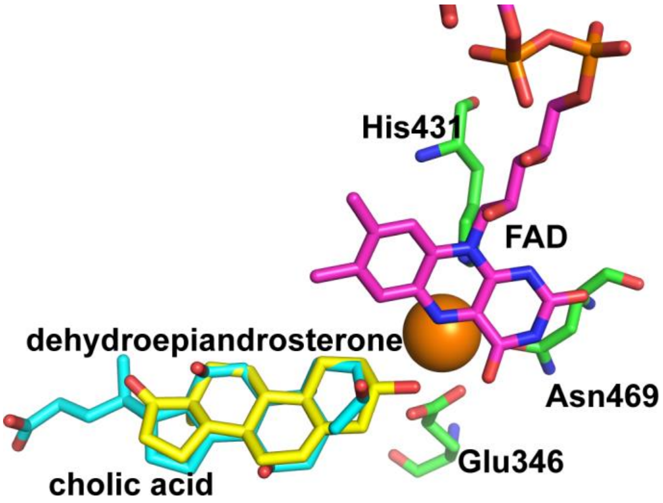

Fig. S4 Comparison of substrate selectivity of PsChO. Cholic acid and dehydroepiandrosterone was colored as cyan and yellow sticks, respectively.

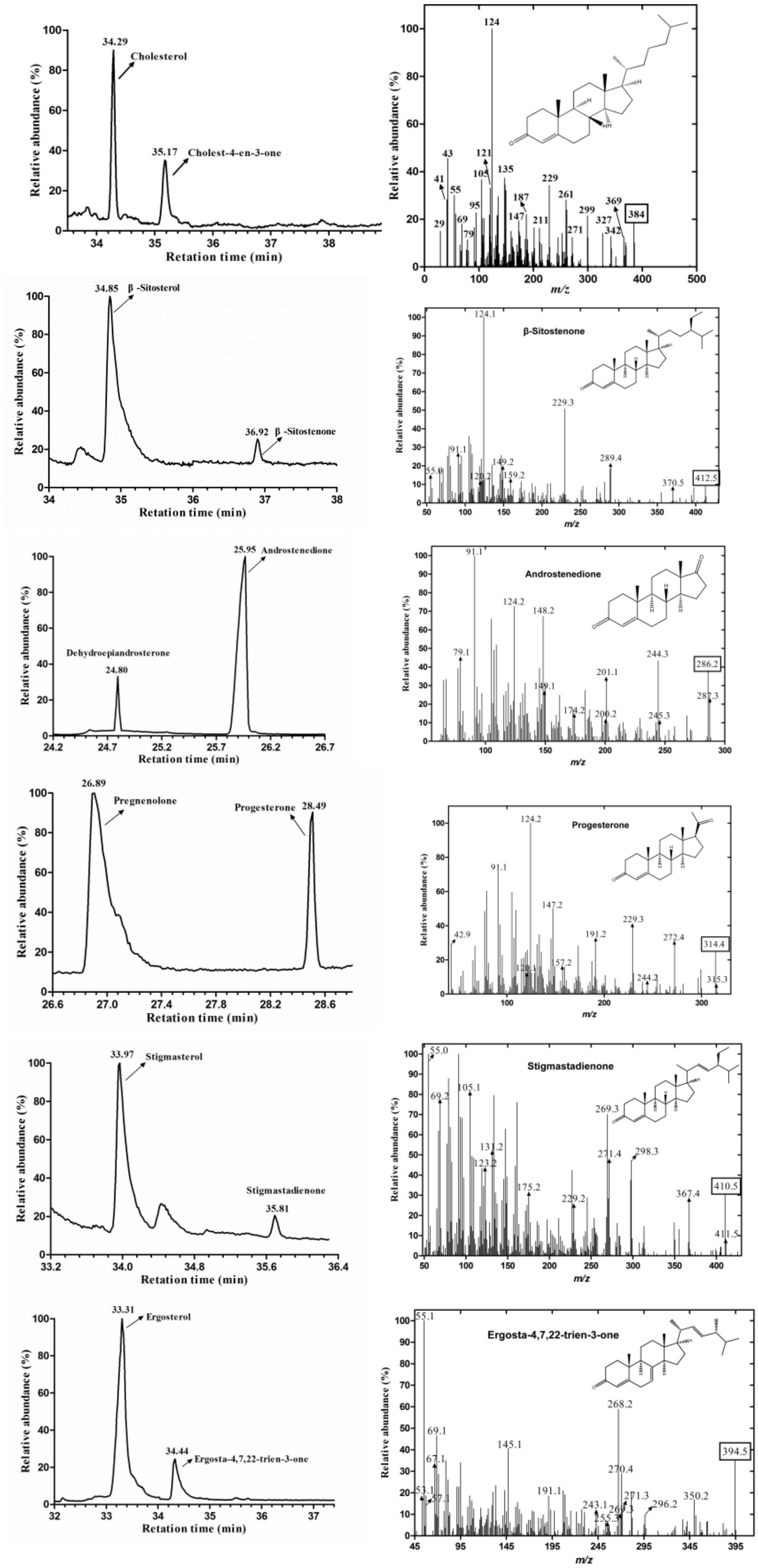

Fig. S5 Products analysis of cholestane skeleton substrates using GC-MS.

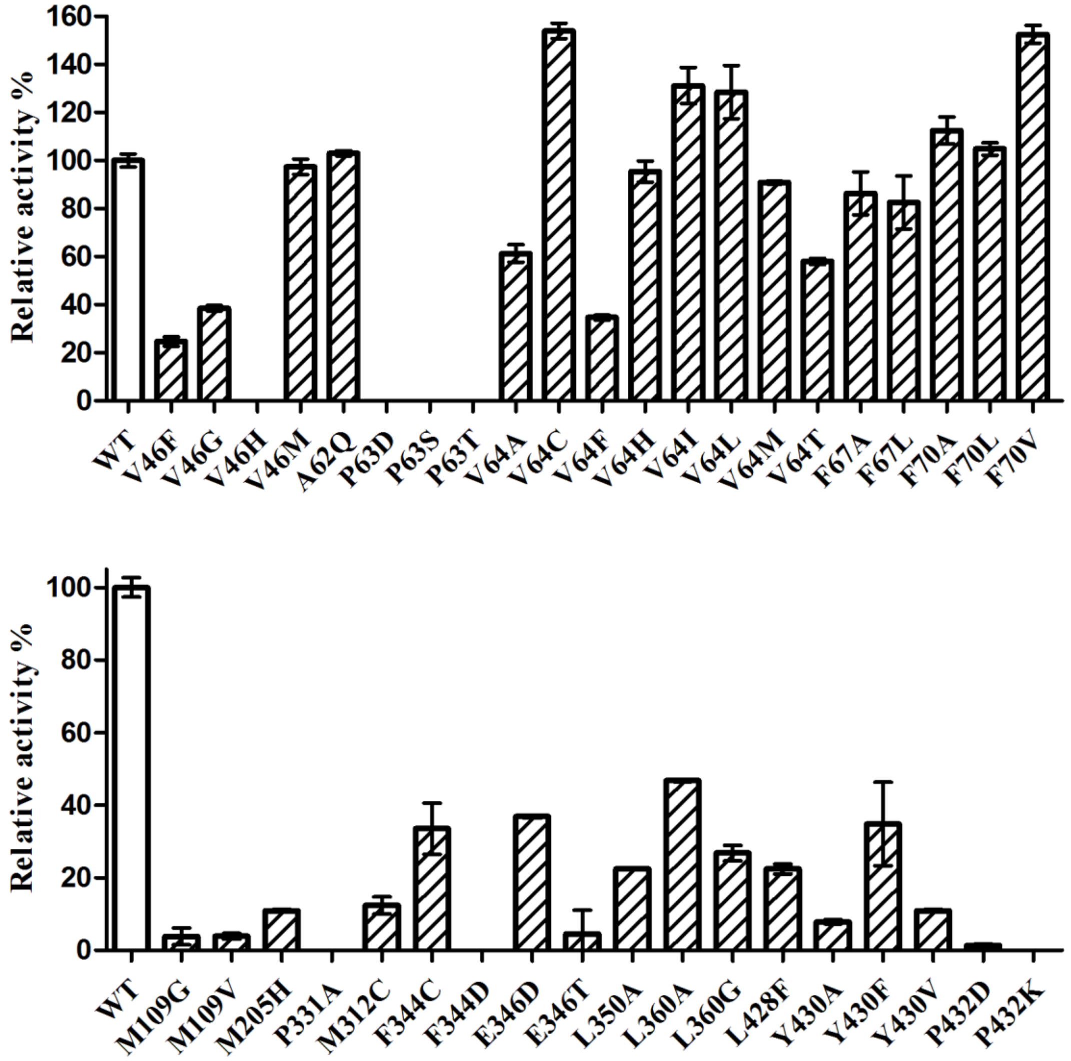

Fig. S6 The relative catalytic activity of PsChO mutants toward cholesterol. The activities of wild-type PsChO are represented as 100 and the error bars are standard deviations (n =3).

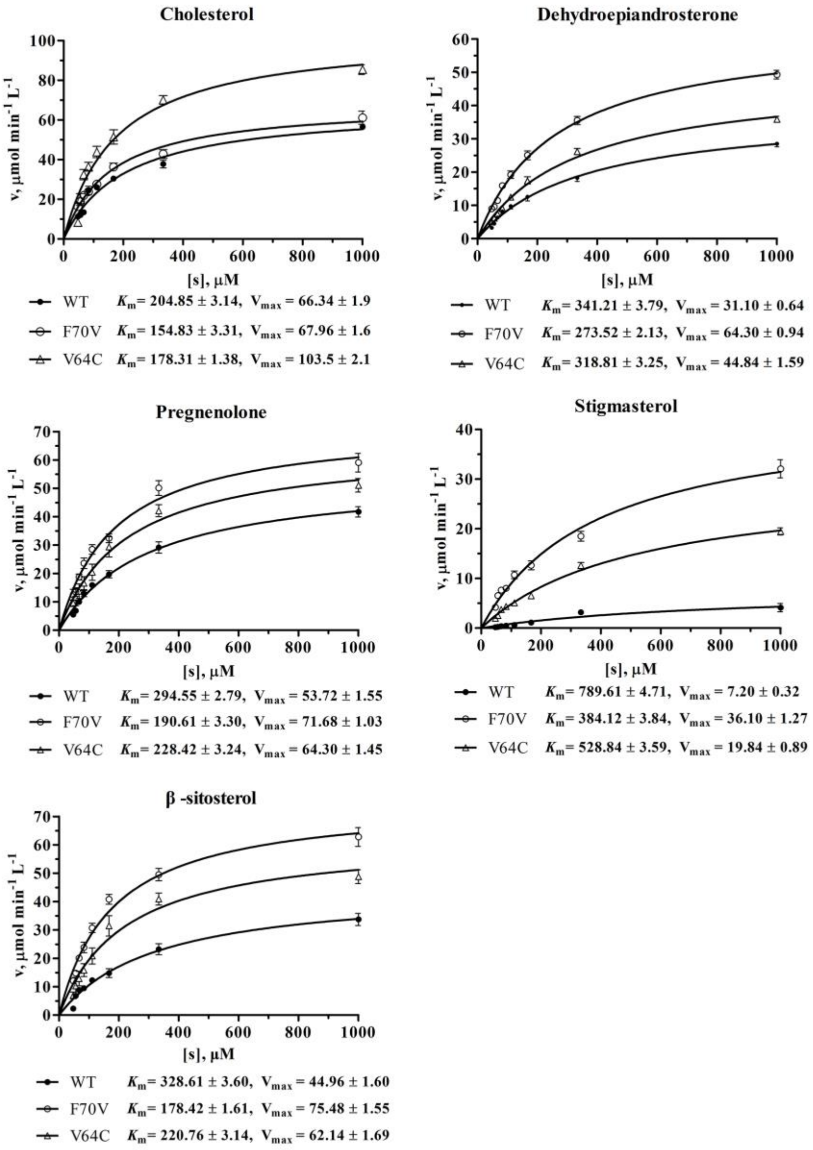

Fig. S7 The fitted curves of initial rate versus substrate concentration plots of PsChO wild-type and F70V, V64C mutants. All assays were repeated three times, and the data are shown as mean ± S.D.

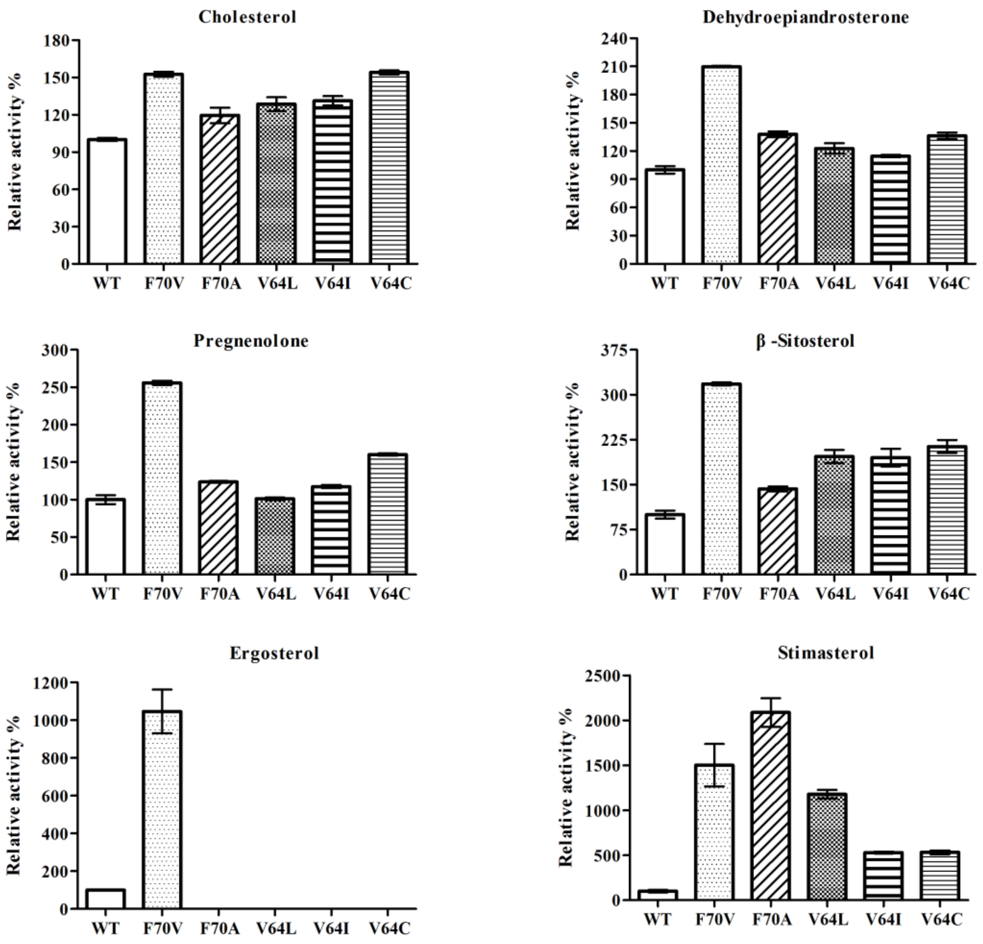

Fig. S8 The relative catalytic activity of PsChO mutants toward various cholestane skeleton substrates. The activities of wild-type PsChO are represented as 100, respectively and the error bars are standard deviations (n =3).

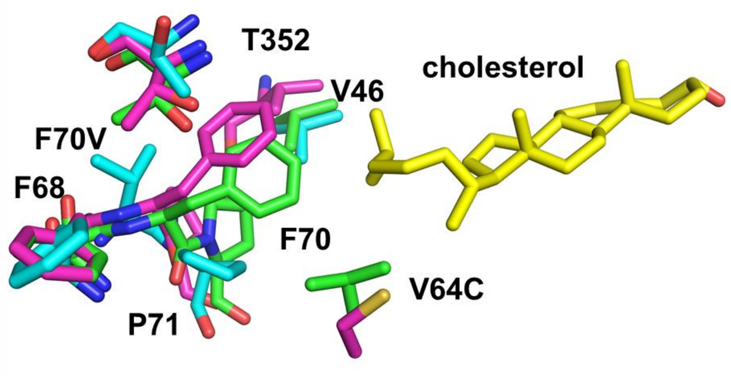

Fig. S9 The superposition of PsChO wild-type and mutants with cholesterol complex using molecular dynamics simulation.
